# Emotional Verbal Fluency: A New Task on Emotion and Executive Function Interaction

**DOI:** 10.3390/bs3030372

**Published:** 2013-07-12

**Authors:** Katharina Sass, Karolina Fetz, Sarah Oetken, Ute Habel, Stefan Heim

**Affiliations:** 1 School of Psychology, University of Queensland, St. Lucia, Brisbane QLD 4072, Australia; 2 Department of Psychiatry, Psychotherapy and Psychosomatics, Medical School, RWTH Aachen University, Pauwelsstr. 30, Aachen 52074, Germany; E-Mails: sheim@ukaachen.de (S.H.); karofetz@googlemail.com (K.F.); soetken@ukaachen.de (S.O.); uhabel@ukaachen.de (U.H.); 3 JARA–Translational Brain Medicine Aachen 52074, Germany; 4 Section Neurological Cognition Research, Department of Neurology, Medical School, RWTH Aachen University, Pauwelsstr. 30, Aachen 52074, Germany; 5 Institute of Neurosciences and Medicine (INM-1), Research Centre Jülich, Jülich 52428, Germany

**Keywords:** verbal fluency, executive functions, emotion, emotion-cognition interaction, semantic

## Abstract

The present study introduces “Emotional Verbal Fluency” as a novel (partially computerized) task, which is aimed to investigate the interaction between emotionally loaded words and executive functions. Verbal fluency tasks are thought to measure executive functions but the interaction with emotional aspects is hardly investigated. In the current study, a group of healthy subjects (n = 21, mean age 25 years, 76% females) were asked to generate items that are either part of a semantic category (e.g., plants, toys, vehicles; standard semantic verbal fluency) or can trigger the emotions joy, anger, sadness, fear and disgust. The results of the task revealed no differences between performance on semantic and emotional categories, suggesting a comparable task difficulty for healthy subjects. Hence, these first results on the comparison between semantic and emotional verbal fluency seem to highlight that both might be suitable for examining executive functioning. However, an interaction was found between the category type and repetition (first *vs*. second sequence of the same category) with larger performance decrease for semantic in comparison to emotional categories. Best performance overall was found for the emotional category “joy” suggesting a positivity bias in healthy subjects. To conclude, emotional verbal fluency is a promising approach to investigate emotional components in an executive task, which may stimulate further research, especially in psychiatric patients who suffer from emotional as well as cognitive deficits.

## 1. Introduction

The ability to control one’s own behavior and to direct one’s actions is an essential ingredient of effective social interactions, and enables us to act self-servingly—a skill that is generally assumed to be part of the executive functions. Executive functions encompass the abilities to make future goals, to plan how to achieve them, to navigate through those plans and to finally monitor and control a successful performance [1]. The testing of executive functions is a common practice in neuropsychological assessment and inferior performance on tasks tapping into executive functioning might be related to lesions or dysfunctions of the frontal cortex [1,2], as well as some psychiatric disorders, e.g., schizophrenia, attention-deficit/hyperactivity disorder or autism [3,4,5]. 

A possibility to investigate executive functions is verbal fluency, which represents the ability to initiate, generate and articulate a word in response to a specific cue and is considered to reflect problem solving abilities [1]. Verbal fluency tasks, such as the Controlled Oral Word Association Test (COWAT; [6]) or the Regensburger Wort-Flüssigkeits-Test (RWT; [7]) are frequently employed during neuropsychological assessment and are used to assess cognitive functioning via associative retrieval and retrieval of words based on phonemic or semantic criteria, respectively. The idea behind this is that brain areas recruited for task solving are crucial and hence, representative of executive functioning. Decreased performance on verbal fluency tasks has been found, for example, in patients with Parkinson’s disease, depression or schizophrenia which have been commonly interpreted as poor executive performance based on frontal dysfunction [8]. 

However, current debates deal with the question of whether verbal fluency tasks are really able to measure “pure” executive functioning, despite the known difficulty to dissociate executive processes from other aspects of cognitive functioning, e.g., intelligence, perceptual speed, attention and working memory [1]. Moreover, not only cognitive but also emotional features might play a role. For example, recent studies showed that emotional information has an influence on semantic processing on behavioral as well as on neural levels, and that both types of information are interconnected, e.g., [9,10,11]. Moreover, Klumpp *et al*. [12] suggested that based on the fact that emotional information in comparison to neutral “may differentially impact frontal lobe functioning, future studies may want to examine verbal fluency for emotional material […]”. 

The experience of emotions is an integral part of human life and it seems to be impossible to separate any situations that are not to a certain degree emotionally loaded. Although it is common in the psychological literature to examine emotion and cognition as two distinct concepts, it is apparent that in real-life human functioning both entities are integrated [13]. The interaction between emotional and cognitive processes has been acknowledged in an ample body of psychological research. Thus, executive functions that are in fact thought to be the essential human ability to control one’s cognitive functions cannot evade the impact of or interaction with concurrent emotional processes. Recent studies [14,15,16,17] found a positive association between mood and semantic networks and performance on verbal fluency tasks, respectively. These findings underline the vulnerability of cognitive performance to induced mood states and consequently, emphasize the necessity to control for such a possible distortion of results through the impact of emotional aspects in neuropsychological assessment. Moreover, beside the shown influence of positive affect on performance during verbal fluency tasks, it is also inevitable to scrutinize to what extent performance might be influenced if emotional material is used. Verbal fluency relies on the fact that every word or concept is linked to associated concepts within a large semantic network, e.g., [18]. However, emotional concepts are thought to be stored in an associational network as well [19,20], leading to the conclusion that emotional information might have a certain impact on the accomplishment of fluency tasks. The fact that emotions and semantics are undoubtedly intertwined (see for example [10] for a detailed discussion) could be also of particular interest when examining executive functions like verbal fluency. In addition, until now there are no studies that investigated the inherent interaction between emotional concepts and cognition in verbal fluency tasks. However, this is especially of interest when examining psychiatric disorders. For example, in their review, Klumpp and Deldin [12] found that earlier studies neither had a closer look on emotional valence in phonemic verbal fluency measures (no information is available if depressed patients produce more negative words for a particular letter) nor are there studies that investigated whether an emotional verbal fluency task might be more sensitive to dorsolateral prefrontal cortex dysregulation in depression. Thus, having knowledge about this might “clarify the degree to which problems inhibiting negative thoughts in depression are due to bilateral disruptions of executive processing” [12]. Hence, the aim of the present study is to introduce and test an adapted version of the verbal fluency tasks—the “Emotional Verbal Fluency” (EmoFlu). ‘EmoFlu’ relies on the idea of investigating the interaction between emotional and executive aspects of cognition that might concurrently influence the performance on verbal fluency tasks. By comparing these processes, the ‘EmoFlu’ task might be the first step to examine the differential influence of neutral *vs*. emotional information on frontal lobe functions as suggested by Klumpp and Deldin [12]. ‘EmoFlu’ is examined by asking subjects to name entities or situations, which might potentially trigger the following five basic emotions: joy, anger, fear, sadness and disgust [21]. Performance on these emotional categories is compared to the subjects’ achievement on a semantic fluency task.

## 2. Method

### 2.1. Participants

Twenty-one healthy subjects (*M*_age_ = 25 years; *SD_age_* = 3; 16 female [76%]) were recruited at the RWTH Aachen University and participated on a voluntary basis. All were native German speakers, had normal or corrected-to-normal vision and were right-handed according to the Edinburgh Inventory of Handedness [22]. Subjects were excluded if they had been diagnosed with past or present neurological/psychiatric diseases or past drug or alcohol abuse. The local ethics committee approved the study and all participants gave informed consent to participate in the study. 

### 2.2. Materials

The design of both tasks was based on a standardized verbal fluency test (Regensburger Wortflüssigkeits-Test [RWT]; [7]). The RWT is a standard test for verbal fluency that is used during neuropsychological (mainly neurological rehabilitation), experimental psychological and pharmapsychological assessments. In general, studies use this test mainly to compare different groups, e.g., old *vs*. young subjects with older subjects showing reduced categorical verbal fluency (e.g., [23]), patients with psychiatric disorders like schizophrenia showing reduced performance of patients in all different word fluency tests (e.g., [24]) or patients with neurodegenerative disorder like Alzheimer who show reduced performance (e.g., [25]). However, the focus of the current study was not to compare different experimental groups but to compare different kind of tasks. 

Prior to the performance of the present experiment, a pilot study with 10 participants was conducted to ensure that the participants understood the instructions and to identify semantic and emotional categories for our purpose. For semantic categories, participants were asked to name single objects or entities belonging to the respective category. For emotional categories, the participants were asked to name all items that could possibly represent one specific emotion. The items could consist of a single object as well as of a short phrase describing certain circumstances, which could elicit the respective emotion. 

Emotional category: On the basis of Izards’s original classification of basic emotions (discussed in [21]), the terms “anger” [Wut], “fear” [Angst], “joy” [Freude], “disgust” [Ekel] and “sadness” [Trauer] were selected as emotional categories. For the sixth basic emotion “surprise”, the pilot testing revealed that it was too difficult for participants to spontaneously mention members of this category, *i.e.*, there was a significant difference between surprise and all other basic emotions, leading to an exclusion of ‘surprise’ as an emotional category. 

Semantic category: Based on the results of the pilot testing, those semantic conditions were chosen that appeared to evoke approximately the same number of items named by the subjects and therefore appeared comparable within category during data analysis. In order to gain comparable data for the semantic and emotional category, five terms were selected as semantic conditions as well: “toys” [Spielzeuge], “vehicles” [Fahrzeuge], “plants” [Pflanzen], “weapons” [Waffen] and “tools” [Werkzeuge].

Randomization of conditions: To prevent any distortion of the data through sequence effects, the order of the 10 different conditions was pseudo-randomized. However, the emotional condition “fear” and the semantic condition “weapons” appeared to be too strongly associated with each other. Therefore, these two conditions were never presented consecutively. Based on this restriction, there were in total eight possible randomizations of the ten conditions (*i.e.*, the five emotional and five semantic conditions). Each participant underwent two randomizations, whereby the order of these two sequences was pseudo-randomized as well. See supplementary material for the exact randomizations and combinations.

### 2.3. Procedure

The procedure was based on the instructions of the RWT, *i.e.*, subjects were asked to avoid repetitions and only name items that exist in the German language [7]. Changes concern the timing (original task = 1 or 2 min once *vs*. adapted tasks = 30 s twice) and that subjects were allowed to use also phrases as response in the adapted tasks.

Every subject sat down in front of a computer screen. Subjects were asked to name members of categories, which would be presented to them. The exact instructions were held constant and appeared on the screen as soon as the experiment was started (see 2.2 Materials for more details). Subjects were alternately presented with either a semantic or an emotional condition—in total there were five of each. The respective condition appeared in the middle of the screen for five seconds, followed by a fixation cross for 30 s. During this period, participants were asked to name as many items as possible that they considered as category members. After 30 s, the fixation cross disappeared and there was a pause of 15 s during which the computer screen appeared blank until the next condition was presented. 

After the first sequence, *i.e.*, five semantic and five emotional conditions, a longer pause of one minute was introduced (blank screen). Subsequently, the sequence was repeated, including the same semantic and emotional conditions but in a different order. The employment of two sequences was used to increase statistical power as well as to control for possible practice effects. Again, the instruction was to name as many items as possible, but now with the restriction to not mention items that had already been stated during the first sequence. The stimulus display was controlled employing a Presentation script file [26]. The responses of the participants were recorded as digital audio files with Audacity (audio editor and recorder [27]) and were simultaneously written down.

### 2.4. Data Analysis

In order to enable participants to associate items freely, no restrictions with regard to their answers were given in the instruction. During the pilot testing and the main experiment it turned out, that semantic conditions were more likely to elicit single-word responses, whereas items named for emotional conditions largely consisted of several words or even small phrases. Thus, the two category types differed with regard to the amount of words and syllables per item, respectively. As participants can name more single-word items than items comprising more syllables, the raw number of correct items appeared to be an inappropriate measure for comparison. It was decided to control for this difference between category types by using the number of items that is corrected for the number of syllables. Consequently, the amount of syllables was counted for every item (N_Syll_per_Item_; e.g., the item *“bicycle*” [Fahrrad] has two syllables), and the mean number of syllables was calculated for every category as a whole across both sequences (M_Syll_per_cat_). In turn, the raw number of correct items (N_Items_per_Cat_) was divided by the mean number of syllables for each category (M_Syll_per_Cat_). The formula would be 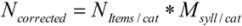. For example, the category “toys” produced 300 correct items across both sequences and all subjects. The sum of syllables for those items is 1027. The number of corrected items N_corrected_ is 3.42 as

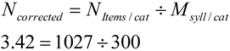



Several statistical analyses were conducted: analysis of variance (ANOVA) with CATEGORY (mean of corrected items for “emotional” and “semantic”) and SEQUENCE (first, second) as within subject factors, ANOVA with CONDITIONS and SEQUENCE as within subjects factors as well as post-hoc paired sample t-tests (corrected for multiple comparisons after Bonferroni) to disentangle significant effects.

The classification procedure of errors is represented in Table 1.

**Table 1:** Error classification.

Type of Error	Explanation	Example	Scoring
(1) Items are not matching the category	The stated item cannot be considered a category member	‘Jungle’ as a response to the semantic category ‘plants’	Item is excluded (error)
(2) Items do not exist	The stated item cannot be found in the German dictionary *	‘Passion fruit-tree’ as response to the semantic category ‘plants’	Item is excluded (error)
(3a) Repetition within the same task sequence	An item is stated twice during responding to a category	-	Item is only counted once as correct (repetition)
(3b) Item represents a superordinate category of other items	One stated item can be considered as a superordinate category of other stated items, which are subordinates	‘Tree’ in response to the semantic category ‘plants’; than naming different kind of trees, e.g., ‘linden’, ‘oak tree’ *etc*.	Superordinate item is excluded; subordinate items are counted as correct (repetition or repetition between task sequence)
(4) Repetition between task sequences	In the second sequence, an item is stated, which has already been mentioned in the first sequence in response to the same category	‘Laugh’ in response to the emotional category ‘joy’ on both the first and the second task sequence	Item is excluded on the second task sequence (repetition between task sequence)

Notes: * Dudenredaktion (Hrsg.): *Duden 01. Die deutsche Rechtschreibung*. 25.Auflage. Band 1, Bibliographisches Institut (Dudenverlag), Mannheim/Wien/Zürich 2009.

Moreover, a brief content analysis of the participants’ response was conducted examining the semantic level of produced items. For example, for the semantic condition “vehicle” most subjects mentioned “bicycle” or “scooter” as response. In contrast, for the emotional category we tried to group the responses according to the situational content, e.g., for the condition “anger” several subjects mentioned situations related to violence or burglary. The idea of this analysis is especially of interest when examining patients with psychiatric disorders (e.g., depression, schizophrenia) as their responses might deviate from the healthy subjects. 

## 3. Results

### 3.1. Error Rates

Error, repetition rates and repetition between sequences rates are presented in Table 2. Incorrect or repeated items were excluded from further analysis. 

**Table 2:** Error and repetition rates per overall category.

	*Semantic*	*Emotional*	*Total*
*Errors*	3.26%	0.29%	2.05%
*Repetition*	3.97%	2.29%	3.29%
*Repetition between sequences*	2.61%	2.48%	2.56%

The comparison between the emotional and semantic categories revealed significant differences in the sum of repetitions (*t*(20) = 4.46, *p* < 0.001) and repetition between sequences (*t*(20) = 2.09, *p* < 0.05) with more repetitions and repetitions between sequences for semantics in comparison to emotions. The sum of errors between both conditions was nearly significant with higher error rates for semantics in comparison to emotions (*t*(20) = 2.01, *p* = 0.06). 

### 3.2. Global Descriptive Statistics

The descriptive statistics for the two category types (raw data and corrected items) and for each of the 10 conditions are presented in Table 3, Table 4.

**Table 3:** Mean number of produced items (raw and corrected data) per category and sequence.

	*Raw data*			Corrected data*			Comparison of sequence effects within category
	*Overall (SD)*	*1^st^ sequence (SD)*	*2^nd^ sequence (SD)*	*Overall (SD)*	*1^st^ sequence (SD)*	*2^nd^ sequence (SD)*	
***Semantic***	15.16 (2.13)	9.70 (1.52)	5.47 (1.14)	51.53 (7.31)	32.91 (5.17)	18.62 (3.90)	*t*(20) = 11.87, *p* < 0.001
***Emotional***	10.69 (2.59)	6.43 (1.53)	4.26 (1.46)	49.89 (12.20)	30.03 (7.14)	19.86 (6.95)	*t*(20) = 6.61, *p* < 0.001
	*t*(20) = 7.78, *p* < 0.001	*t*(20) = 8.97, *p* < 0.001	*t*(20) = 3.34, *p* < 0.005	*t*(20) = 0.65, *p* = 0.52	*t*(20) = 1.89, *p* = 0.07	*t*(20) = 0.79, *p* = 0.44	

**Table 4:** Mean number of items (per task sequence and in total; raw and corrected data) and standard deviations.

	*Raw data*			Corrected data *		
*Category*	*1^st^ sequence (SD)*	*2^nd^ sequence (SD)*	*Overall (SD)*	*1^st^ sequence (SD)*	*2^nd^ sequence (SD)*	*Overall (SD)*
*Toys*	10.71 (1.90)	5.52 (1.69)	16.24 (2.10)	36.68 (6.51)	18.91 (5.79)	55.59 (7.17)
*Vehicles*	10.90 (2.39)	5.33 (1.96)	16.24 (2.39)	33.04 (7.23)	16.16 (5.93)	49.20 (7.22)
*Plants*	10.10 (2.34)	7.24 (2.53)	17.33 (4.42)	35.01 (8.13)	25.10 (8.77)	60.11 (15.33)
*Weapons*	8.33 (2.44)	4.43 (2.04)	12.76 (3.6)	30.52 (8.92)	16.22 (7.47)	46.74 (13.20)
*Tools*	8.43 (2.16)	4.81 (2.52)	13.24 (3.49)	29.31 (7.50)	16.72 (8.77)	46.03 (12.14)
*Anger*	5.48 (1.78)	3.81 (2.23)	9.29 (3.64)	28.02 (9.10)	19.49 (11.40)	47.51 (18.60)
*Fear*	6.76 (1.92)	4.71 (1.68)	11.48 (3.01)	29.46 (8.37)	20.54 (7.31)	50.00 (13.11)
*Joy*	7.71 (2.43)	5.67 (2.06)	13.38 (3.5)	35.07 (11.06)	25.76 (9.35)	60.83 (15.91)
*Disgust*	6.38 (2.09)	3.90 (1.97)	10.29 (3.52)	29.49 (9.64)	18.05 (9.12)	47.54 (16.28)
*Sadness*	5.81 (2.42)	3.19 (2.29)	9.00 (4.01)	28.12 (11.71)	15.44 (11.10)	43.56 (19.42)

Notes: * raw number of correct items x mean number of syllables for each category = corrected items/SD = standard deviation.

### 3.3. Overall Comparison of Both Main Conditions

The ANOVA with CATEGORY and SEQUENCE as within-subject factors revealed a significant influence of sequence (*F*(1, 20) = 133.76, *p <* 0.001) with less items produced in the second sequence in comparison to the first sequence (31.48 *vs*. 19.24). This was also true for each category alone (see Table 2 for the exact statistics). The influence of CATEGORY was not significant (*F*(1, 20) = 0.43, *p =* 0.52), *i.e.*, there were no differences between the semantic and emotional category overall. However, a significant interaction of CAGEGORY*SEQUENCE (*F*(1, 20) = 5.36, *p <* 0.05) was found as subjects performance decreased more for the semantic category (Sequence 1 – Sequence 2 = 14.29) than for the emotional category (Sequence 1 – Sequence 2 = 10.17). This performance change differed significantly between the semantic and emotional category (*p* < 0.05). However, the performance between the emotional and semantic category within one sequence did not differ but showed a trend for the first sequence with less items being produced for emotions (*M*_semantic_ = 32.92 *vs*. *M*_emotional_ = 30.03; *p* = 0.07). 

### 3.4. Comparison of Each Single Condition

The ANOVA with CONDITIONS and SEQUENCE as within-subject factor indicated significant differences between the ten CONDITIONS (*F*(9, 180) = 4.91, *p <* 0.001) and a significant interaction of CONDITIONS*SEQUENCE (*F*(9, 180) = 2.51, *p <* 0.01) with differences for each condition between the sequences.

Post-hoc paired samples t-tests revealed significant differences between some conditions (independent of sequence) with most items being produced for “plants” and fewest for “sadness”. When corrected for multiple comparisons, differences were found between “vehicles” and “plants” (*p* < 0.05), “plants” and “tools” (*p* < 0.05) and “joy” and “disgust” (*p <* 0.05*)*. In addition, all conditions showed significant differences between the first and second sequence with less items being produced in the second sequence.

When comparing the different semantic conditions within each task sequence, the results for the first sequence revealed that most items were produced for “toys” and fewest for “tools”. For the second sequence, significant differences were found between plants and all other categories. Most items were produced for “plants”, and fewest were for “vehicles” (see Table 5).

**Table 5:** Within category comparison for semantics.

Comparison	1^st^ sequence		2^nd^ sequence	
	*t*(20) =	*p <*	*t*(20) =	*p <*
toys > weapons	3.27	0.005	n.s.	n.s.
toys > tools	3.31	0.005	n.s.	n.s.
plants > vehicles	n.s.	n.s.	4.03	0.001
plants > weapons	2.11	0.05	3.55	0.005
plants > tools	2.97	0.01	3.27	0.005
plants > toys	n.s.	n.s.	2.78	0.05
vehicles > tools	2.51	0.05	n.s.	n.s.

Notes: n.s. = not significant.

For the emotional category, the t-tests revealed that all conditions differed significantly from “joy” in the first and second sequence. In addition, for the second sequence there was also a significant difference between “fear” and “sadness”, with more items being produced for “fear”. In the first sequence, most items were produced for “joy”, fewest for “anger”. For the second sequence, again “joy” elicited most items; fewest were “sadness” (see Table 6). 

**Table 6:** Within category comparison for emotions.

Comparison	1^st^ sequence		2^nd^ sequence	
	*t*(20) =	*p <*	*t*(20) =	*p <*
joy > anger	2.64	0.05	2.60	0.05
joy > fear	2.98	0.01	2.23	0.05
joy > disgust	2.60	0.05	3.21	0.005
joy > sadness	2.37	0.05	3.77	0.001
fear > sadness	n.a.	n.a.	2.24	0.05

### 3.5. Descriptive Content Analysis

For the content analysis, the raw data were counted for the semantic category, *i.e.*, not the syllables but the entire word. In contrast, for the emotional category, different entities and/or situations were put into one single data point as most of the time different types of phrases were mentioned (e.g., for the condition “joy” one subject said “present” while another one said “receiving a present”). To assess the amount of variation, the number of participants that named the same item was counted (*i.e.*, over 50% or at least 11 of 21 subjects named the same item and over 75% or at least 16 of 21 subjects named the same item). In addition, to assess the variability of the remaining items, the number of different items was compared with the number of overall raw items produced per category.

Semantic category: Only small variations in answers could be found and several items could be identified which were named by over 50% or even over 75% of the participants for a respective category. For example, “bicycle” and “scooter” were named as “vehicles”, “rose” as “plant” and “knife”/“gun” as “weapons” by at least 16 of 21 subjects. However, some categories were more common than others. To evaluate variations the number of items that was produced by less than 25% of the subjects was counted. The results showed that “vehicles” produced the most equal items (38% of items were below 25%), followed by “tools” (40%), “toys” (48%), “plants” (50%) and “weapons” (52%) with the highest variability. 

Emotional categories: Overall, answers were more individual but due to the naming format they were collapsed into groups. In other words, similarities in responses across healthy subjects were not observed regarding the exact items but with respect to the semantic cluster these items belong to. For example, situations related to

- violence (e.g., affray, fight) or to burglary (e.g., to steal, breaking and entering) were named for the condition “anger”,
- dangerous animals (e.g., toxic snakes, sharks, dogs) or examination (e.g., exam nerves, test) were named for the condition “fear”,
- benefit/victory (e.g., winner of the world championship, win the lottery) or, “party/celebrate” (e.g., to celebrate, go clubbing) were named for the condition “joy”,
- specific food (e.g., Brussels sprout, cauliflower) or “excretion” (e.g., shit, urine), for “disgust”, and
- funeral (e.g., laying out, grave) for “sadness”.

Comparing the variety within the group of emotions (*i.e.*, number of items produced by less than 25% of the subjects), “disgust” (22%) showed the most similar responses, followed by “fear” (26%) and “joy” (25%), “sadness” (33%) and with the highest variability for “anger” (57%) due to the naming of single personal situations and memories.

The content analysis can be found in the supplementary material (Table A1).

## 4. Discussion

The present paper had the aim to introduce a new task called ‘EmoFlu’ which is based on the idea that emotional information might have an influence on verbal fluency performance and that there might be an interaction between emotional and executive aspects of cognition. Decreased performance on verbal fluency tasks is typically attributed to an individuals’ executive dysfunction. However, the concept of ‘EmoFlu’ assumes that emotional concepts have an impact on fluency tasks and that an emotion-processing deficit might concurrently impair patients’ performance on tests of executive functions. ‘EmoFlu’ was tested by asking subjects to name entities or situations that potentially trigger the emotions “joy”, “anger”, “sadness”, “disgust” and “fear” whereby differences between the performance on ordinary semantic fluency tasks and emotional verbal fluency were of special interest. 

The results of the first behavioral measurements showed that subjects exhibited a low rate of errors and repetitions, which implies that the instructions were well understood and could be followed. Interestingly, the sum of errors showed a trend with more errors being made for the semantic in comparison to the emotional category. The cause might be that the semantic category comprises rather concrete items and criteria (e.g., “bicycle” is a vehicle while “doll” is not) while the emotional category is formed by personal experiences and memories, *i.e.*, rather abstract items (e.g., some people are afraid of spiders other not). Hence, the overall sum of error might not be the best way to evaluate the emotional fluency task because of the individuality of responses in the subjects.

No overall differences in the performance of subjects were found between the semantic and emotional categories overall when corrected for number of syllables (and independent of sequence). This result implies comparable difficulty of both category types, although the conditions within one category differed from each other. However, both the semantic and emotional categories seem to be suitable instruments for examining verbal fluency in healthy individuals with an equal overall performance. 

A closer look on the emotional category revealed that subjects performed by far best on the emotional condition “joy” compared to all other emotional condition. A limitation of the current design is the use of four negative emotions and one positive emotion. To control for effects, we averaged the corrected overall performance for the negative emotions and compared it with the performance for the positive emotion in a post-hoc test. The result still showed that performance on “joy” was significantly better than for negative emotions (*p* < 0.001). This finding supports a large body of research suggesting that healthy individuals generally exhibit a positivity bias, which affects many aspects of cognition, e.g., [28,29]. For example, during studies on automatic semantic processing it was shown that subjects process positive information easily and comparable to neutral information while negative information is suppressed or even inhibited, e.g., [10]. In addition, as the task is not only based on emotional processing but also on cognitive processes, the presentation of a emotionally negative word might “interfere” with the cognitive processes leading to inferior performance (see also [10] for a discussion of emotional interference with cognitive processes). Moreover, subjects use words of positive valence more often than negative words (“Pollyanna effect”; [30]) and more easily retrieve positive category members than negative ones [31]. Overall, in line with earlier studies the results of the present study showed that healthy subjects easily “imagine” situations that elicit positive feelings and thereby perform best on the emotional condition “joy”. This result also supports the suggestion that positive and negative emotional materials are differentially organized in memory where positive information might be better elaborated and interconnected than negative [10,20,32]. However, as one anonymous reviewer pointed out the performance of subjects on the two tasks might also be influenced by different memory systems (*i.e.*, episodic *vs*. semantic memory), for example, the influence of episodic memory on emotional verbal fluency might be stronger compared to semantic fluency. However, with the current study we were not able to distinguish between both. In addition, recent studies showed that semantic and episodic memory might be interactive rather than completely separable memory systems (e.g., [33,34]). For example, Ryan *et al*. showed that generation and recalling items from various categories involves strategies like retrieval of autobiographical and spatial contextual information. In other words, the authors suggested that episodic information may be used to generate semantic material. Furthermore, following the definition of Tulving [35], episodic memory is experiential in nature and related to details of time and place. Robinson and Clore [36] further defined ‘emotional episodic memory’ as “knowledge about one’s emotions in a particular place at a particular time”. Following these definitions, EmoFlu might only partially involve episodic memory recall because, for example, ‘giving birth’ was declared for the condition “joy”, but the subject did not have a baby, or that ‘plane crash’ was mentioned for “fear” but the person had never experienced one. To conclude, we agree that there might be specific influences of semantic and episodic memory on both categories. However, with the current setting it is impossible to disentangle both effects, and this would go beyond the focus of the current study. Future studies might therefore ask subjects after the experiment how they generated the items of the different categories to examine if there is an interaction of both memory systems or if there are qualitative differences between them. 

Beside the type of category, the task sequence had an influence on results. Both categories showed performance decreases during the second sequence. On the one hand, this could be caused by the current instruction. Like in the original version of the RWT, subjects were asked not to repeat items that were already mentioned before, *i.e.*, possible memory (recall) and inhibition processes might be involved. On the other hand, the instruction was held constant for both conditions but the decrease in performance was more pronounced for semantic conditions than for emotional conditions. One possible reason might be practice effects. For example, less performance changes were found mainly for emotional conditions (lowest change for “anger”, followed by “fear” and joy”). Maybe subjects needed more time to become used to the task and get used to the kind of emotional categories (more “open” than semantic categories). In accordance with this suggestion, further examination of the interaction between category type and task sequence revealed that on the first task sequence subjects showed a trend to perform better on the semantic than on the emotional category (*p* = 0.07). On the second task sequence, performance on both category types was nearly similar. Hence, it could be suspected that more time is needed to perform properly on emotional categories whereas subjects are able to cope with semantic categories directly from the beginning. It appears that the training during the first sequence of the task is especially effective for emotional categories—even if the performance overall decreases in the second sequence. To conclude, although further research is needed to examine the origin of the influence of task sequence on task performance, the existence of an interaction itself underlines the necessity to conduct two or even more task sequences. Furthermore, when employing the task in parallel with traditional verbal fluency tests, this might help to separate the contribution of emotional and executive aspects on task performance. In addition, it may have clinical applicability to investigate if patients that exhibit emotion-processing deficits (e.g., schizophrenic or depressive patients) perform worse than healthy subjects on semantic fluency tasks but even disproportionately poor on the ‘EmoFlu’ task which explicitly relies on emotional components, this might suggest that the patient’s emotional disturbances are “responsible” for the differential impairment in semantic and emotional verbal fluency tasks.

The limitations of the current study are that the collection of the first behavioral data was primarily of explorative nature. It has to be strongly emphasized that the results drawn from the small sample of participants have to be treated with caution and should be regarded as a trend which can serve as a starting point for the extension for further studies. It would be interesting to further investigate whether the results found for healthy subjects will be confirmed in the extension of the study and also to examine how psychiatric patients will perform on the task as patients may show emotion processing deficits that can impact the performance. The question is if EmoFlu is able to help to understand the nature of this impairment and its impact on cognition as there are some problems comparing both verbal fluency tasks. However, following the model of Bower [20] who suggested an associative network of memory and emotion, emotional states can be represented as nodes in a semantic network, *i.e.*, each emotion is represented by a specific node and “the arousal of an […] emotion spreads activation through the network of associations surrounding that [...] emotion” [37]. Support arises from studies showing that positive and negative words are processed differently, for example, during automatic semantic priming [10,38] and that patients with depression show disturbed (behavioral and neural) processing of emotional concepts influenced by their current mood [38]. Hence, the suggestion is that patients with emotion-processing deficits have problems to execute EmoFlu because of their distinct processing of emotional concepts within the semantic association network. However, the interaction of emotion and cognition in clinical populations is more complex than in healthy subjects that makes an investigation of emotion-cognition-interaction difficult and should be carefully controlled.

In addition, it is known that age has an influence on the performance in verbal fluency tasks while gender differences have no influence [39]. However, gender differences occur for emotional processing and recollection of memory (autobiographical/emotional memory), e.g., [40,41]. Hence, future studies might have a look on the influence of age and gender on EmoFlu. One last limitation is the current randomization. While the condition “weapons” and “fear” did not follow each other, there might be other associations between semantic conditions and emotional evaluations (e.g., “fruit” and “joy”) as earlier studies using paradigms like the Go/NoGo association ask have shown, e.g., [43]. 

## 5. Conclusions

The present study had the aim to introduce the new task ‘EmoFlu’ and to elaborate on the rationale behind this concept. Data showed that there is an influence of emotion on executive functions highlighting the strong correlation between both concepts. The ‘EmoFlu’ task could act as an important tool in neuropsychological assessment, serving as an instrument for emotional aspects of cognition that are not yet considered in verbal fluency tasks. 

## Supplementary

**Table A1:** Brief content analysis of produced items.

	Toys	Vehicles	Plants	Weapons	Tools
> 25 % (at least 5 from 21 subjects)	-Car -Bobby car -Board game -Duplo (Lego) -Toy train -Playing cards -Spinning top -Stuffed animal -Memory -Playmobil -Marbles -Dollhouse -Puzzle -Teddy -Drum	-Excavator -Bobbycar -Bus -Tricycle -Unicycle -Carriage -Moped -Armor -Rollerblades -Ship -Skateboard -Streetcar -Subway -Train	-Apple tree -Pear tree -Beech -Ivy -Oak -Daisy -Cactus -Cherry tree -Common dandelion -Daffodil -Carnation -Orchid -Palm tree -Fir tree -Viola	-Ax -Dagger -Artillery shell -Machine gun -Saber -Brass knuckles -Bat -Sword	-Ax -Rasp -Chisel -Knife -Nail -Scissors -Percussion drill
> 50 % (at least 11 of 21 subjects)	-Ball -Building bricks -Lego -Doll	-Car -Airplane -Truck -Motorcycle	-Grass -Sunflower -tulip	-Rifle	-Saw -Pair of tongs
> 75 % (at least 16 of 21 subjects)	n.a.	-Bicycle -Scooter	-Rose	-Knife -Gun	-Drill -Hammer -Screwdriver
	*Anger*	*Fear*	*Happy*	*Disgust*	*sadness*
> 25 % (at least 5 from 21 subjects)	-Trouble -Cheating -Shouting -Burglary -Violence -Lying -Conflict -Inequity -Loss -To fail	-Loneliness -Narrowness -Plane crash -Height -Horror -Disease -Crowd -New situations -Violence -Loud noise -Spider -Death -Accident -Loss -To fail	-Appreciation -Visit -Success -Family -Free time -Birthday -Love -Nice weather -Beach -Surprise -Christmas	-Disease -Blood -Sanies -Vomiting -Bugs/insects -Waste -Wounds -Fungi -Mucus -Spider -(dirty) toilet -Spoiled food	-Loneliness -Farewell -Breakup -Accident -
> 50 % (at least 11 of 21 subjects)	n.a.	-Darkness -Dangerous animals -Examination	-Benefit/victory -Pass an exam -Children/birth -Holiday -Party/ celebrate	-(specific) Food -Dejection	-Funeral
> 75 % (at least 16 of 21 subjects)	n.a.	n.a.	-Presents -Friends	-Dirt -Malodor	-Death -Loss
